# Tie2 Signaling Cooperates with TNF to Promote the Pro-Inflammatory Activation of Human Macrophages Independently of Macrophage Functional Phenotype

**DOI:** 10.1371/journal.pone.0082088

**Published:** 2014-01-03

**Authors:** Samuel García, Sarah Krausz, Carmen A. Ambarus, Beatriz Malvar Fernández, Linda M. Hartkamp, Inge E. van Es, Jörg Hamann, Dominique L. Baeten, Paul P. Tak, Kris A. Reedquist

**Affiliations:** 1 Department of Experimental Immunology, University of Amsterdam, Amsterdam, the Netherlands; 2 Department of Clinical Immunology and Rheumatology, University of Amsterdam, Amsterdam, the Netherlands; University of Leuven, Rega Institute, Belgium

## Abstract

Angiopoietin (Ang) -1 and -2 and their receptor Tie2 play critical roles in regulating angiogenic processes during development, homeostasis, tumorigenesis, inflammation and tissue repair. Tie2 signaling is best characterized in endothelial cells, but a subset of human and murine circulating monocytes/macrophages essential to solid tumor formation express Tie2 and display immunosuppressive properties consistent with M2 macrophage polarization. However, we have recently shown that Tie2 is strongly activated in pro-inflammatory macrophages present in rheumatoid arthritis patient synovial tissue. Here we examined the relationship between Tie2 expression and function during human macrophage polarization. Tie2 expression was observed under all polarization conditions, but was highest in IFN-γ and IL-10 –differentiated macrophages. While TNF enhanced expression of a common restricted set of genes involved in angiogenesis and inflammation in GM-CSF, IFN-γ and IL-10 –differentiated macrophages, expression of multiple chemokines and cytokines, including *CXCL3*, *CXCL5*, *CXCL8*, *IL6*, and *IL12B* was further augmented in the presence of Ang-1 and Ang-2, via Tie2 activation of JAK/STAT signaling. Conditioned medium from macrophages stimulated with Ang-1 or Ang-2 in combination with TNF, sustained monocyte recruitment. Our findings suggest a general role for Tie2 in cooperatively promoting the inflammatory activation of macrophages, independently of polarization conditions.

## Introduction

The tyrosine kinase receptor Tie2 makes essential contributions to vascular development and blood vessel remodeling through its interaction with angiopoietin (Ang) ligands, of which Ang-1 and Ang-2 are the best characterized [1], [2]. Ang-1 binding to Tie2 induces autophosphorylation of Tie2 on multiple tyrosine residues and activation of downstream signaling pathways. Tie2 signaling has been most extensively studied within the context of endothelial cell (EC) biology and vascular development and homeostasis. Ang-1 promotes Tie2-dependent EC survival, stability of the endothelial barrier, vascularization, and lymphangiogenesis [3]–[5]. The outcome of Ang-1 signaling to ECs is context-dependent, as *trans* signaling of Tie2 via Ang-1 presented by adjacent ECs strengthens endothelial barriers, while Ang-1 deposited on extracellular matrix components promotes EC proliferation and migration [6], [7]. Overexpression of Ang-2 during development antagonizes Ang-1 function [1], [2]. Ang-2 can compete with Ang-1 to prevent phosphorylation of Tie2, and this antagonistic effect is most readily observed in blocking Tie2 activation at EC cell-cell junctions [7], [8]. In the absence of Ang-1, or when Ang-2 is present in high concentrations, Ang-2 can stimulate Tie2 signaling [8]. Ang-2 can also initiate EC signaling cascades via direct binding to integrins, as evidenced by the ability of Ang-2 to promote sprouting angiogenesis of Tie2-negative ECs *in vitro* and *in vivo* [9]. Ang-2 effects on the vascular are often pro-inflammatory, promoting vascular leakage and sensitizing ECs to tumor necrosis factor (TNF)-dependent induction of adhesion protein expression important for leukocyte extravasation [10]–[12].

Myeloid cells expressing Tie2 also make requisite contributions to angiogenic processes in health and disease. A subpopulation of circulating monocytes and tumor-associated macrophages (TAMs) express Tie2 in humans and mice [13], [14]. Tie2-expressing monocytes (TEMs) are recruited to solid tumors in the earliest stages of tumor development, and targeted depletion studies in mice have shown that TEMs are needed to allow vascularization and growth of solid tumors [13]. TEMs represent a small subset of the total human peripheral blood mononuclear cell (PBMC) population, are readily detected in human tumors, and human tumor xenograft models indicate that TEMs play similar pro-angiogenic, pro-tumorigenic roles in both mice and humans [14]. Gene expression analyses of murine TEMs suggest that they represent a distinct committed myeloid lineage derived from embryonic myeloid cells and circulating monocytes [15]. Ang-2 stimulation serves to recruit TEMs and induce TEM expression of pro-angiogenic genes such as *TP* and *CTSB* [13], [14], [16]. Gene expression analyses also suggest a relationship between TEMs and polarized macrophages with immunosuppressive/immunoregulatory phenotypes [15], [17]. Indeed, Ang-2 induces interleukin (IL)-10 expression in TEMs, promoting the expansion and suppressive capacity of regulatory T cells [17].

The phenotypic characteristics and functional capacities of macrophages are tightly regulated by environmental cues they receive during differentiation, a process referred to as macrophage polarization. Cytokines and pathogens, among other factors, can guide macrophage differentiation into (classically activated) pro-inflammatory M1 or (alternatively activated) M2 wound-healing and immunosuppressive macrophages [18]. *In vivo*, tissues can be populated by both M1- and M2–like macrophages, as well as macrophages of mixed phenotype. To date, TEMs have been described to display M2-like properties in mice and humans [15], [17]. However, we have recently found that Tie2 is highly activated in synovial macrophages of patients with rheumatoid arthritis (RA) [19]. In RA, pro-inflammatory activation of these macrophages is very closely linked with disease activity and patient response to therapy [20]. These observations suggested that Tie2 function in myeloid biology may not be limited to immunosuppressive functions associated with TEMs, and in the present work, we examined the relationship between Tie2 expression and function in human macrophages within the context of macrophage polarization.

## Materials and Methods

### Monocyte purification and macrophage differentiation

Human PBMCs were isolated from volunteer donor blood buffy coats (Sanquin) by gradient centrifugation with Lymphoprep (Axis-Shield PoPAS) and monocytes were further isolated by Percoll gradient separation (GE Healthcare). Monocytes were differentiated into macrophages in Iscove's modified Dulbecco's medium/10% fetal calf serum supplemented with 100 µg/ml gentamycin (Invitrogen), in the presence of granulocyte-macrophage colony-stimulating factor (GM-CSF, 5 ng/ml), macrophage colony-stimulating factor (M-CSF, 25 ng/ml), IL-4 (10 ng/ml), interferon (IFN)-γ (10 ng/ml) or IL-10 (10 ng/ml) (all from R&D Systems) for 7 d.

### Flow cytometry

Macrophage purity, differentiation and Tie2 expression was assessed by flow cytometry (FACS Canto Flow Cytometer, BD Biosciences). Fluorochrome-labeled monoclonal antibodies against CD16 (clone 61D3, eBiosciences), CD64 (Biolegend), CD163, CD200R (both from BD Pharmingen) and Tie2 (R&D Systems), and equivalent concentrations of isotype control antibodies, were used. Before staining, Fc receptors were blocked with 10% human serum (Lonza). Data were analyzed with Flow Jo Flow Cytometry Analysis software (Tree Star). Values were expressed as the ratio of the geometric mean fluorescence intensity (geomean) of the marker of interest over that of the isotype control.

### RT-PCR and quantitative (q)PCR arrays

Macrophages were left unstimulated or were stimulated with TNF (10 ng/ml, Biosource International) in the presence or absence of recombinant human Ang-1 or Ang-2 (200 ng/ml, R&D Systems) for 4 h. Where indicated, macrophages were preincubated for 1 h in the absence or presence of the Janus kinase (JAK) inhibitor AG490 (10 µM, Calbiochem-Merck). RNA was isolated using the RNeasy Kit and RNase-Free DNase Set (Qiagen). Total RNA was reverse-transcribed using SuperScript™ II RT (Invitrogen). Duplicate PCR reactions were performed using SYBR green (Applied Biosystem) with an ABI Prism® 7000 sequence detection system (Applied Biosystems). cDNA was amplified using specific primers (Tie2 forward (for), 5′-3′, ACAATGGTGTCTGCCATGAA; Tie2 reverse (rev), TTCACAAGCCTTCTCACACG; Tie1 for, CAGATTGCGCTACAGCTAGG; Tie1 rev, CCGCGTAAGTGAAGTTCTCA; IL-12B, ACGTTTCACCTGCTGGTGGCT; IL-12B rev, CTCCGCACGTCACCCCTTGG; IL-6 for, GACAGCCACTCACCTCTTCA; IL-6 rev, CCTCTTTGCTGCTTTCACAC; GAPDH for, GCCAGCCGAGCCACATC; GAPDH rev, TGACCAGGCGCCCAATAC, all from Invitrogen). Relative levels of gene expression were normalized to GAPDH housekeeping gene. The relative quantity (RQ) of mRNA was calculated by using the formula: 2^−ΔΔCt^. Alternatively, total RNA was subjected to cDNA synthesis using the RT^2^ First Strand Kit (Qiagen) and mRNA expression of 84 angiogenic factors was analyzed by RT-PCR using low density qPCR Array (Human Angiogenic Growth Factors & Angiogenesis Inhibitors PCR Array, Qiagen). Relative levels of gene expression were normalized to 5 housekeeping genes and RQ values determined as above.

### Measurement of cytokine production

Macrophages were stimulated 24 h with TNF (10 ng/ml) in the presence or absence of Ang-1 or Ang-2 (200 ng/ml). Cell-free supernatants were analyzed by ELISA for IL-6, IL-10 IL-8 and thrombospondin (TSP)-2 (PelKine Compact™ ELISA kits, Sanquin Reagents). CXCL-5, -6, CCL-3, -7, and IL-12B were measured using human single-plex assays (Bio-Rad) and read on a Bio-Plex 200 system (Bio-Rad).

### Immunoblotting

Macrophages total cell lysates were subjected to electrophoresis on 4–12% gradient Bis-Tris SDS NuPAGE® gels (Invitrogen), and proteins were transferred to polyvinylidene membranes (Millipore). Membranes were incubated overnight at 4°C in primary antibodies specific for nuclear factor of kappa light polypeptide gene enhancer in B-cells inhibitor alpha (IκBα), protein kinase B (PKB), phospho(p)-PKB (Ser473), extracellular signal-regulated kinase (ERK), p-ERK, p38, p-p38, and histone 3 (H3) (all from Cell Signalling), washed, and incubated in TBS/T containing horseradish peroxidase–conjugated secondary antibody. Protein was detected with Lumi-light^plus^ Western Blotting Substrate (Roche Diagnostics) using an ImageQuant LAS 4000 system (GE Healthcare). Densitometry analysis was performed with Image J software. Relative protein phosphorylation or expression was normalized to total protein or H3, respectively.

### Monocyte chemotaxis assay

Macrophages were stimulated with TNF in the presence or absence of Ang-1 or Ang-2 for 24 h and cell-free supernatants were collected. Monocytes were purified from human PBMCs using Monocyte Isolation Kit II (Miltenyi Biotec). 1×10^5^ monocytes were transferred into the upper chamber of 5 µm pore-size transwell plates (96 well ChemoTX®, NeuroProbe). Fresh medium alone, or containing Ang-1 or Ang-2 (200 ng/ml), or conditioned macrophage supernatants were added to the lower chamber. After 2 h at 37°C, cells migrating to the lower chamber were quantified by staining with Calcein-AM (1 µM, BD Bioscience) and analysis using a multi-label reader Victor3™ (PerkinElmer Inc.). Data were expressed as signal (arbitrary units), following subtraction of signal of empty wells containing no cells.

### Measurement of STAT family member DNA binding

Nuclear fractions of macrophages were isolated using Nuclear Extract Kit (Active Motif) and DNA binding activities of STAT-1, -3, -5A, and -5B were determined using a TransAM transcription factor ELISA (Active Motif) according to the manufacturer's instructions.

### Statistical analyses

Statistical analysis was performed using Windows GraphPad Prism 5 (GraphPad Software, Inc.). Potential differences between experimental groups were analyzed by non-parametric, Kruskal-Wallis test or Friedman test, as appropriate. P-values <0.05 were considered statistically significant.

## Results

### Human macrophage Tie2 expression is regulated by polarizing cytokines but is not restricted to immunoregulatory M2 macrophages

**Figure 1:**
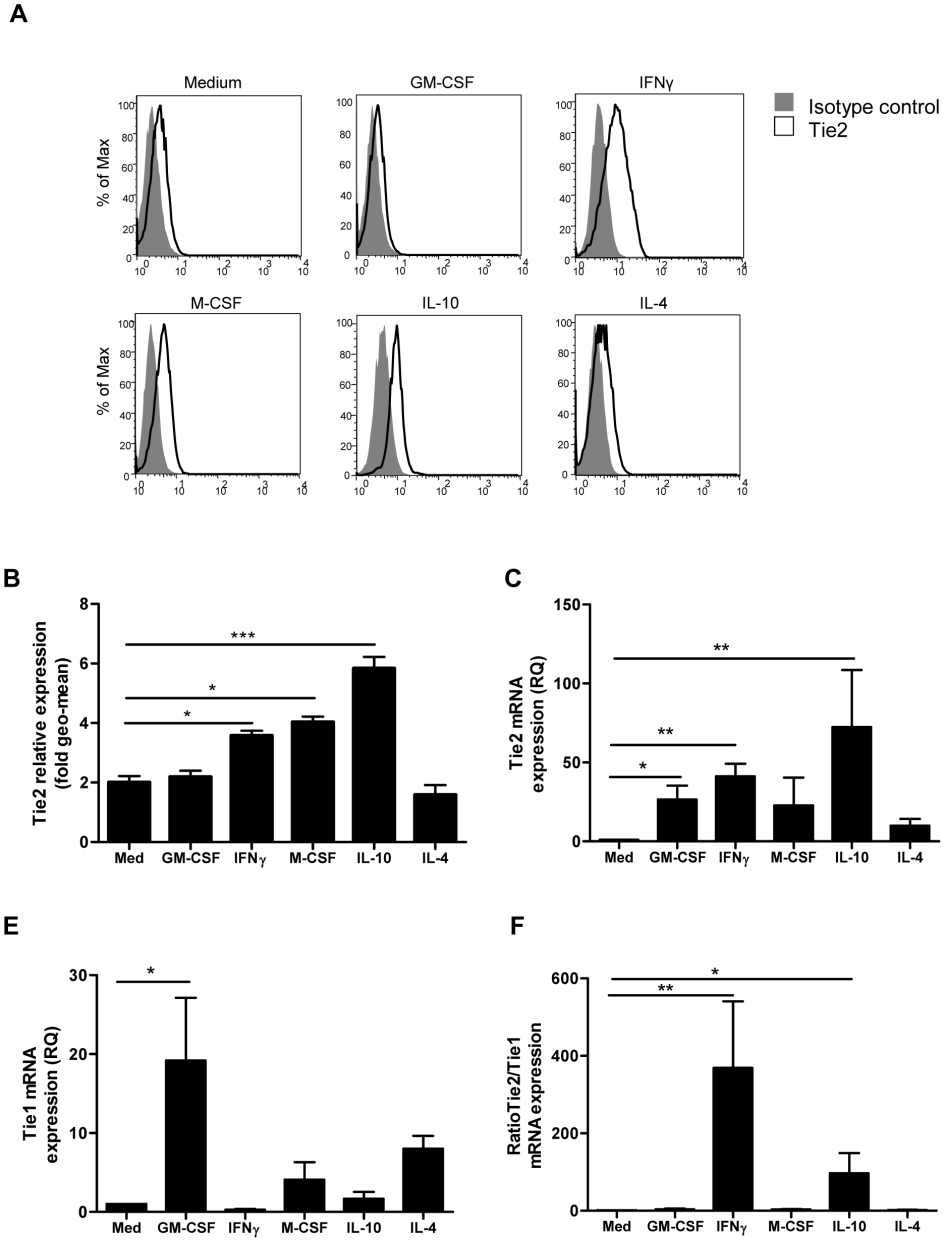
Regulation of Tie2 and Tie1 expression during macrophage polarization. (A) FACS analysis of macrophages polarized for 7 d with the indicated cytokines, using Ig control (filled grey area) and Tie2 antibodies (open area). Experiments shown are representative of 7 independent experiments. (B) Quantification of relative Tie2 surface expression (geomean) and Tie2 mRNA expression (C) by macrophages differentiated in medium alone or with the indicated cytokine. (D) Quantification of relative Tie1 mRNA expression and (E) Tie2 mRNA expression relative to Tie1 by macrophages differentiated in medium alone or with indicated cytokine. qPCR data is shown as relative quantity, as described in material and methods Values are the mean ± SEM of 5–7 independent experiments. ^*^P<0.05. ^**^P<0.01 versus macrophages differentiated in medium alone. Kruskal-Wallis test was used for statistical analyses.

**Figure 2:**
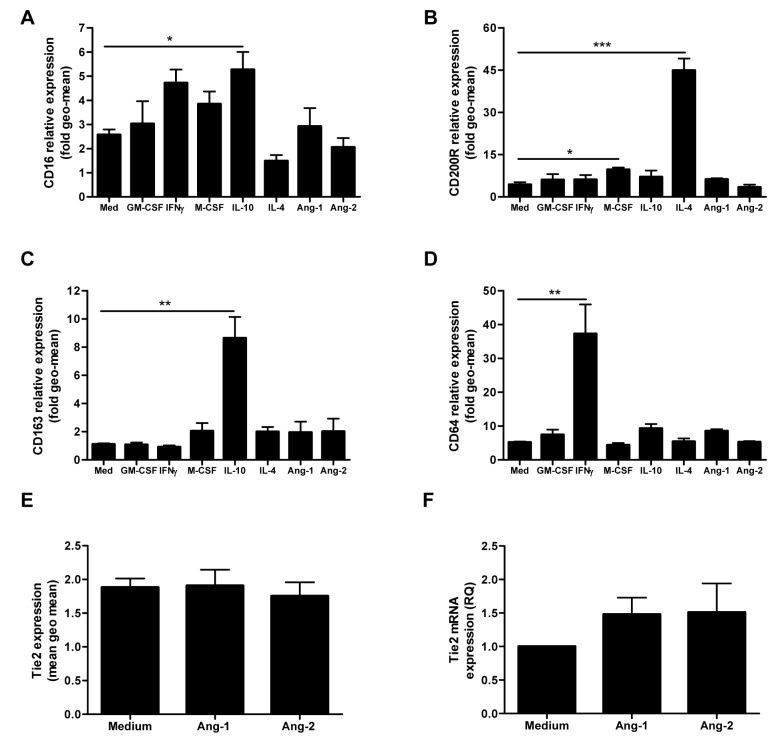
Ang-1 and Ang-2 fail to induce macrophage polarization. (A–D) FACS analysis of expression of macrophage surface markers (A) CD16, (B) CD163, (C) CD200R and (D) CD64 in macrophages differentiated for 7 d in medium alone, or medium containing the indicated polarizing cytokines, Ang-1 (200 ng/ml) or Ang-2 (200 ng/ml). Data in A–D are presented as the geomean normalized to values obtained for macrophages cultured in medium alone, and represent the mean ± SEM of 4–5 independent experiments per marker. (E) Surface protein expression (geomean) and mRNA expression (F) of Tie2, as determined by FACS analysis (n = 5) and qPCR (n = 3), respectively, in human macrophages cultured for 7 d in the absence (medium) or presence of 200 ng/ml Ang-1 or Ang-2. *P<0.05, **P<0.01, ***P<0.001, versus macrophages differentiated in medium alone. Kruskal-Wallis test was used for statistical analyses.

To determine the influence of polarization on macrophage Tie2 expression, we differentiated macrophages in the presence of cytokines resulting in the generation of pro-inflammatory M1 macrophages (GM-CSF, IFN-γ) or wound healing/immunoregulatory M2 macrophages (M-CSF, IL-4 and IL-10) [18], [21]. FACS analysis of polarized macrophages revealed that although each cytokine differentially regulated Tie2 surface expression, there was no relationship between Tie2 expression and M1 or M2 macrophage functional classification (Figure 1A). Compared to medium alone, IFN-γ, M-CSF and IL-10 significantly enhanced macrophage Tie2 surface expression, while little Tie2 was detected on macrophages exposed to IL-4 (Figure 1A–B). Tie2 mRNA expression mirrored results obtained in examining protein expression, with the highest levels of Tie2 mRNA expressed in macrophages differentiated in IFN-γ (MΦ_IFN_), IL-10 (MΦ_IL10_) and GM-CSF (MΦ_GM-CSF_) (Figure 1C). Expression of Tie1, reported to act as an antagonist of Tie2 signaling in ECs, was also differentially induced in macrophages during polarization. Compared to medium alone, GM-CSF significantly enhanced Tie1 mRNA expression, and trends toward enhanced expression were observed in macrophages differentiated in M-CSF (MΦ_M-CSF_) and IL-4 (MΦ_IL4_) (Figure 1D). Tie2 mRNA expression relative to Tie1 was significantly enhanced in MΦ_IFN_ and MΦ_IL10_ compared to medium alone (Figure 1E). FACS analysis of recently validated human macrophage polarization markers CD16, CD64, CD163 and CD200R [22], [23] confirmed that macrophage culture conditions resulted in the polarization of macrophages with expected phenotypic characteristics (Figure 2A–D). Thus, in human macrophages, while macrophage polarization conditions regulate Tie2 and Tie1 expression levels, Tie2 expression is not limited to M2-like macrophages.

### Ang-1 and Ang-2 do not influence macrophage polarization

We next examined the possibility that Ang-1 and Ang-2 might directly regulate macrophage polarization, as acute stimulation of freshly derived human monocytes with Ang-2 can further enhance Tie2 expression and reinforce immunoregulatory gene expression patterns [16]. Macrophages were differentiated in the absence or presence of Ang-1 or Ang-2 and specific markers of polarized macrophages were analyzed by flow cytometry. Unlike classical polarizing cytokines, neither Ang-1 nor Ang-2 influenced expression of CD16, CD64, CD163, or CD200R, surface markers strongly up-regulated by IFN-γ, IL-10 and IL-4, respectively (Figure 2A–D) [22], [23]. Additionally, neither Ang-1 nor Ang-2 significantly altered Tie2 protein or mRNA expression (Figure 2E–F). Together, these data observed suggest that macrophage Tie2 signaling is not sufficient to drive human macrophage polarization or initiate feedback loops regulating Ang or Tie2 expression.

### Ang-1 and Ang-2 regulate cytokine production in M1 and M2 macrophages

**Figure 3:**
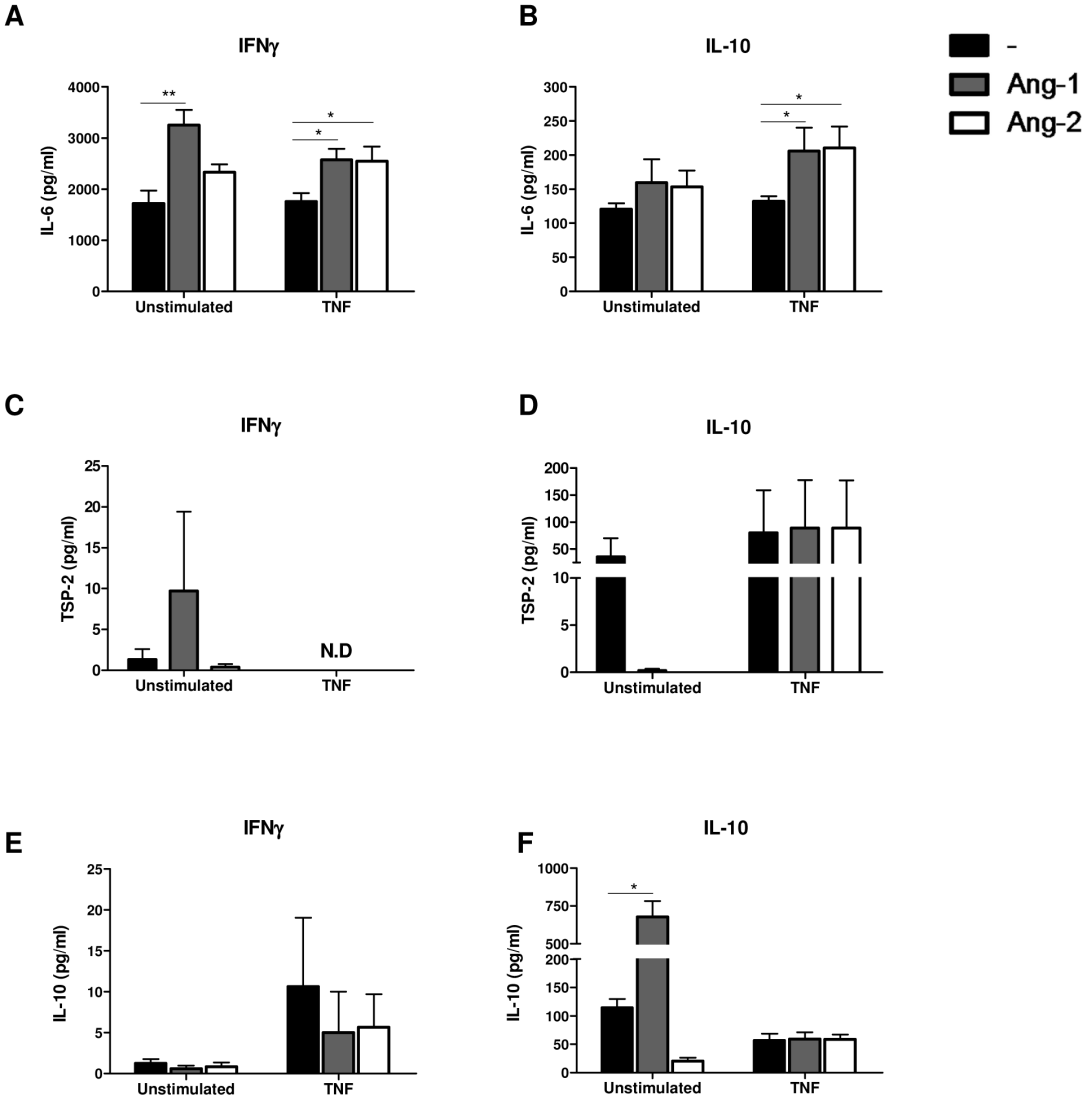
Ang-1 and Ang-2 regulation of cytokine production in polarized macrophages. Analyses of (A,B) IL-6, (C,D) TSP-2, and (E,F) IL-10 production in supernatants of macrophages differentiated in (A,C,E) IFN-γ or (B,D,F) IL-10 after 24 h incubation in medium alone or TNF-α (10 ng/ml) in the absence (white bars) or presence of Ang-1 (200 ng/ml, gray bars) or Ang-2 (200 ng/ml, black bars). Bars represent the means and SEM of 4–7 independent experiments. N.D., not detectable. *P<0.05, **P<0.01 versus cells not exposed to Ang-1 or Ang-2. Friedman test was used for statistical analyses.

We next examined the influence of Tie2 signaling on macrophage cytokine production. MΦ_IFN_ and MΦ_IL10_ were stimulated in the absence or presence of Ang-1 or Ang-2, alone or in combination with TNF. In MΦ_IFN_, levels of IL-6 production were elevated by Ang-1 in the absence of TNF (P<0.01) and by Ang-1 and Ang-2, in the presence of TNF (P<0.05 in both cases) (Figure 3A). In MΦ_IL10_, both Ang-1 (P<0.05) and Ang-2 (P<0.05) enhanced TNF-induced IL-6 production (Figure 3B). We also analyzed expression of the anti-angiogenic factor TSP-2, as we had previously demonstrated that Ang-2, but not Ang-1, suppressed TSP-2 production in MΦ_GM-CSF_) [19]. TSP-2 levels were low in unstimulated MΦ_IFN_, but were not influenced by Ang-1 or Ang-2, and stimulation of these cells with TNF suppressed TSP-2 production below detection levels (Figure 3C). Trends were observed toward suppressed spontaneous TSP-2 production by MΦ_IL10_ in the presence of either Ang-1 or Ang-2 (Figure 3D). We also considered the influence of Ang-1 and Ang-2 on immunoregulatory cytokine IL-10, as previous work has shown that Ang-2 increased IL-10 production in TEMs [16]. In MΦ_IFN_, neither Ang-1 nor Ang-2 influenced IL-10 production by themselves or following stimulation with TNF (Figure 3E). However, in MΦ_IL10_, Ang-1 significantly enhanced IL-10 production, while a trend was observed towards suppressed IL-10 production in the presence of Ang-2 (Figure 3F). Together, based on the parameters assessed, these data fail to indicate a general immunosuppressive role for Ang-1 and Ang-2 in the biology of differentiated macrophages *in vitro*.

### Ang-1 and Ang-2 enhance macrophage expression of TNF-induced chemokines and pro-inflammatory cytokines

**Figure 4:**
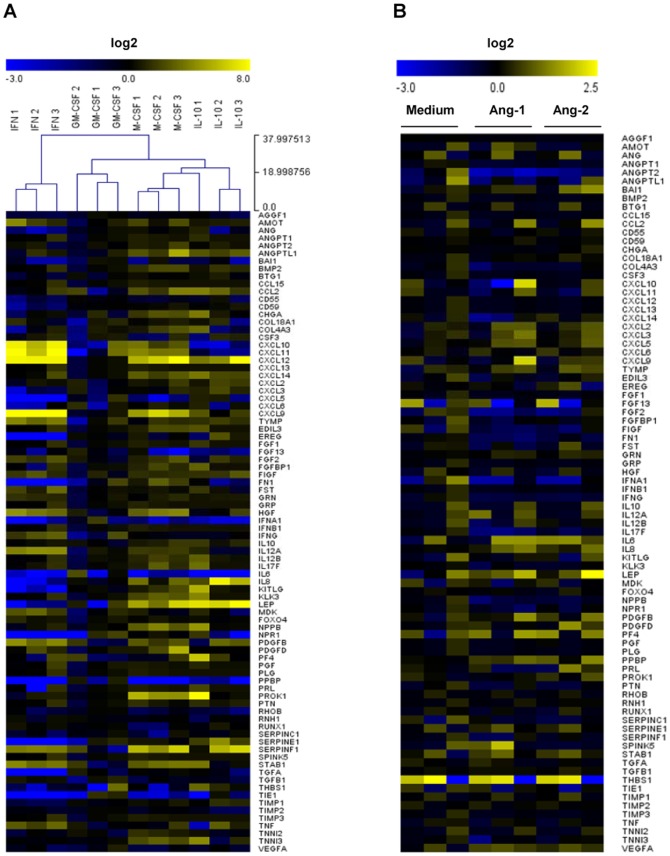
Macrophage polarization influences angiogenic expression profile. (A) mRNA expression profiles of 84 angiogenesis related genes in macrophages differentiated in GM-CSF, M-CSF, IFN-γ and IL-10 7 d (n = 3). Data is presented as an unsupervised clustergram. (B) Heat map analysis of mRNA levels of angiogenesis-related genes in macrophages differentiated in GM-CSF after 4 h incubation in medium alone or Ang-1 (200 ng/ml) or Ang-2 (200 ng/ml).

To gain further insight into the effects of Tie2 signaling on inflammatory properties of polarized macrophages, we explored the effects of macrophage stimulation with Ang-1 and Ang-2 on mRNA expression of 84 genes involved in angiogenic processes. MΦ_IFN_, MΦ_GM-CSF_, MΦ_M-CSF_, and MΦ_IL10_ displayed distinct profiles of gene expression, although unsupervised clustering of data revealed the highest relationship of gene expression patterns between M1 MΦ_IFN_ and MΦ_GM-CSF_, and between M2 MΦ_M-CSF_ and MΦ_IL-10_ (Figure 4A). Results obtained were in general agreement with previously published studies of gene expression during macrophage polarization as, for example, MΦ_IFN_ displayed the highest expression of *CXCL9*, *CXCL10*, *CXCL11*, and *IL12A* (Figure 4A) [24].

**Figure 5:**
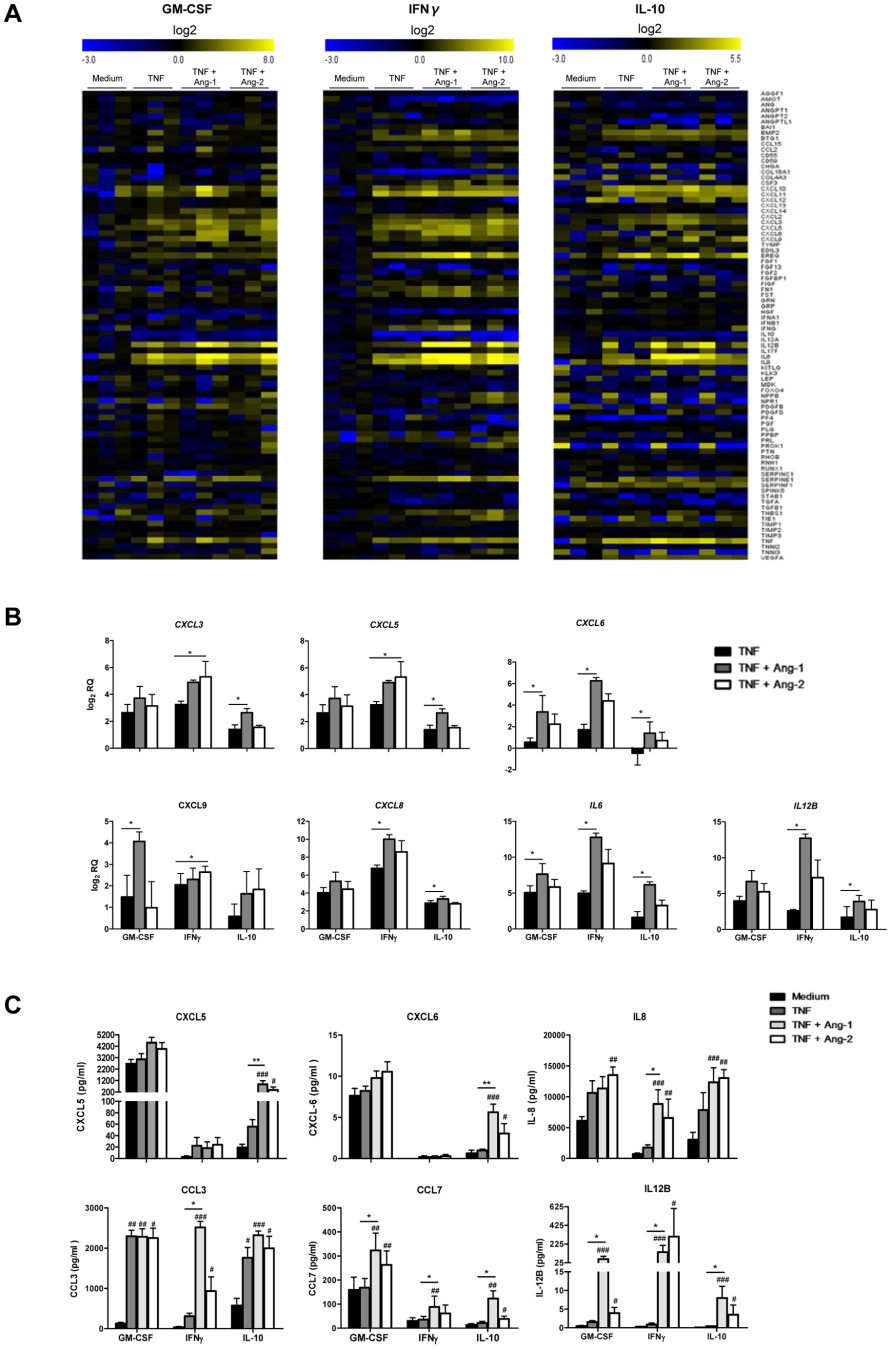
Ang-1 and Ang-2 enhance the TNF-induced expression of chemokines and cytokines. (A) mRNA expression profiles angiogenesis related genes in macrophages differentiated with GM-CSF, IFN-γ or IL-10 after 4 h incubation in medium alone or TNF-α (10 ng/ml) in the absence or presence of Ang-1 (200 ng/ml) or Ang-2 (200 ng/ml) (n = 3). Data is presented as a heat map where lowest mRNA expression is showed in dark blue and highest in yellow. (B) Analyses of chemokines and cytokines mRNA expression levels of selected genes analyzed in (A). Data is shown as log_2_ relative quantity respect to unstimulated cells, as described in material and methods. Bars represent the means and SEM of 3 independent experiments. *P<0.05, between stimulatory conditions. Friedman test was used for statistical analyses. (C) Multiplex analysis of protein production by macrophages differentiated in GM-CSF, IFN-γ, or IL-10 after 24 h incubation in medium alone or TNF (10 ng/ml) in the absence or presence of Ang-1 (200 ng/ml) or Ang-2 (200 ng/ml). Bars represent the means and SEM of 6 independent experiments. *P<0.05, * P<0.01, between stimulatory conditions, #P<0.05, ##P<0.01, ### P<0.001, compared to unstimulated cells. Friedman test was used for statistical analyses.

We chose MΦ_GM-CSF_, a commonly studied macrophage, and M1 MΦ_IFN_ and M2 MΦ_IL-10_, as three functionally distinct types of macrophages expressing high levels of Tie2, for further analysis. Surprisingly, we were unable to identify additional genes which were reproducibly regulated by more than 2-fold in these macrophages following stimulation with either Ang-1 or Ang-2 alone (Figure 4B and data not shown). However, recent studies have indicated that while Ang-1 and Ang-2 are relatively weak regulators of gene expression in ECs and macrophages, these cytokines can cooperate in a synergistic fashion with TNF to regulate inflammatory gene induction [12], [19]. We observed that of the 84 genes examined, 24 were induced by at least 2-fold by TNF in MΦ_IFN_ in 3 independent experiments, 19 in MΦ_GM-CSF_, and 20 in MΦ_IL10_ (Figure 5A). Interestingly, of the 30 different genes induced by TNF in the 3 polarization conditions, 11 were induced in all 3 macrophage types, although quantitative differences in basal and induced gene expression levels were readily apparent. When macrophages were stimulated with Ang-1 or Ang-2 in combination with TNF, we observed that Ang-1 and Ang-2 primarily influenced the subset of genes already regulated independently by TNF: 17 of 27 in MΦ_IFN_, 11 of 19 in MΦ_GM-CSF_, and 11 of 20 in MΦ_IL10_ (Figure 5A and Table S1). Many of these genes were regulated in multiple types of macrophages. Ang-1 cooperated with TNF to significantly (P<0.05) increase mRNA levels of *CXCL6*, *CXCL9*, and *IL6* in MΦ_GM-CSF_, *CXCL6*, *CXCL8*, *IL6* and *IL12B* in MΦ_IFN_, *CXCL3*, *CXCL5*, *CXCL6*, *CXCL8*, *IL6* and *IL12B* in MΦ_IL10_. Ang-2 cooperated with TNF (P<0.05) to induce *CXCL3*, *CXCL5*, and *CXCL9* in MΦ_IFN_ (Figure 5B). This synergism was independent of the polarization conditions, although the effects of Ang-1 and Ang-2 were most robust in MΦ_IFN_. In both MΦ_IFN_ and MΦ_IL10_, a dose-dependent cooperation of Ang-1 and Ang-2 (2–200 ng/ml) with TNF in the induction of *IL6* and *IL12B* was observed (data not shown). Other genes were targeted by Ang-1 in a polarization-restricted fashion, such as *CXCL2* in MΦ_IL10_, and *BMP2, EREG, SerpinE1 and BTG1* in MΦ_IFN_ (Figure S1A and Table S1). In almost all cases Ang-1 was more effective than Ang-2 in cooperating with TNF to induce gene expression (Figure 5B and Table S1), but in MΦ_IFN_ we identified a small subset of genes, *CHGA*, *IL17F*, *NPPB*, *PROK1* and *Tie1*, which were upregulated only in the presence of TNF in combination with Ang-2 (Figure S1B).

We next sought to confirm the effects of Ang-1 and Ang-2 on TNF-induced gene expression in polarized macrophages at the protein level, selecting a subset of secreted products. Of the analytes assessed, only CCL-3 was significantly induced by TNF, in MΦ_GM-CSF_ and MΦ_IL10_. However addition of Ang-1 in combination with TNF significantly induced secretion of CCL-7 and IL-12B in MΦ_GM-CSF_, IL-8, CCL-3, CCL-7, and IL-12B in MΦ_IFN_, and CXCL-5, CXCL-6, IL-8, CCL-3, CCL-7, and IL12-B in MΦ_IL10_. Ang-2 cooperated with TNF to significantly induce production of IL-8, CCL-7, and IL-12B in MΦ_GM-CSF_, IL-8, CCL-3, and IL-12B in MΦ_IFN_, and CXCL-5, CXCL-6, IL-8, CCL-3, CCL7, and IL-12B in MΦ_IL10_ (Figure 5C). Together these results suggest that Ang-1 and Ang-2 play highly overlapping roles in cooperating with TNF to induce pro-inflammatory cytokines and chemokines, independently of macrophage polarization conditions.

### Ang-1 and Ang-2 cooperate with TNF to promote monocyte recruitment by macrophages

**Figure 6:**
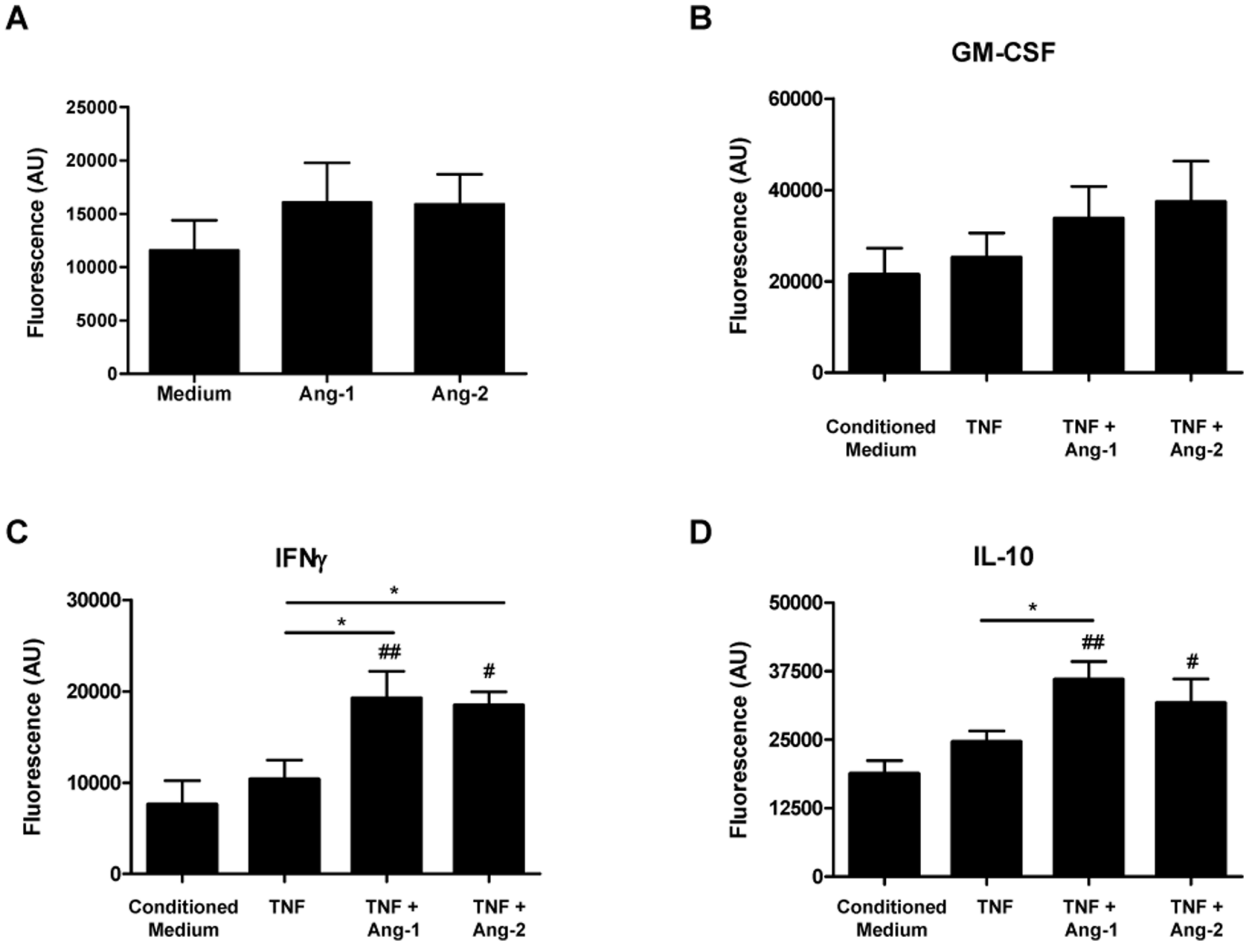
Ang-1 and Ang-2 stimulation of macrophages cooperates with TNF to induce monocyte recruitment. Monocyte migration assays in response to (A) chemotaxis buffer alone (medium) or in combination with Ang-1 (200 ng/ml) or Ang-2 (200 ng/ml), or chemotaxis buffer supplemented with conditioned medium of macrophages polarized in (B) GM-CSF, (C) IFN-γ or (D) IL-10 in the absence (unstimulated) or presence of 24 h stimulation with TNF (10 ng/ml) alone or in combination with Ang-1 (200 ng/ml) or Ang-2 (200 ng/ml). Bars represent the means and SEM of 7–8 independent experiments. Experiments with each polarization condition were performed in parallel, but for ease of analysis are presented as 3 independent graphs. *P<0.05, * P<0.01, between stimulatory conditions, #P<0.05, ##P<0.01, ### P<0.001, compared to conditioned medium. Friedman test was used for statistical analyses.

To determine if the effects we observed of Ang-1 and Ang-2 on macrophage chemokine production were sufficient to influence biological responses, we performed monocyte chemotaxis assays. In control experiments, inclusion of Ang-1 or Ang-2 alone in chemotaxis buffer resulted in a trend towards induced monocyte migration, which did not reach statistical significance (Figure 6A). Additionally, inclusion of TNF alone or in combination with Ang-1 or Ang-2 in conditioned medium from MΦ_GM-CSF_, MΦ_IFN_, or MΦ_IL10_ failed to influence monocyte chemotaxis compared to conditioned medium alone (data not shown). Conditioned medium from macrophages stimulated with TNF increased monocyte migration compared to conditioned medium from control macrophages, although differences were not statistically significant (Figure 6B–D). Conditioned medium from macrophages stimulated with TNF in combination with Ang-1 induced significantly higher migration compared to medium from unstimulated macrophages, and macrophages stimulated with TNF alone in MΦ_IFN_ (P<0.05) and MΦ_IL10_ (P<0.01). Ang-2 also enhanced TNF-induced monocyte migration compared to control conditioned medium (P<0.05) in MΦ_IFN_ and MΦ_IL10_, and compared to TNF alone in MΦ_IFN_ (P<0.05).

### Ang-1 and Ang-2 increase TNF-induced cytokine production via JAK/STAT signaling

**Figure 7:**
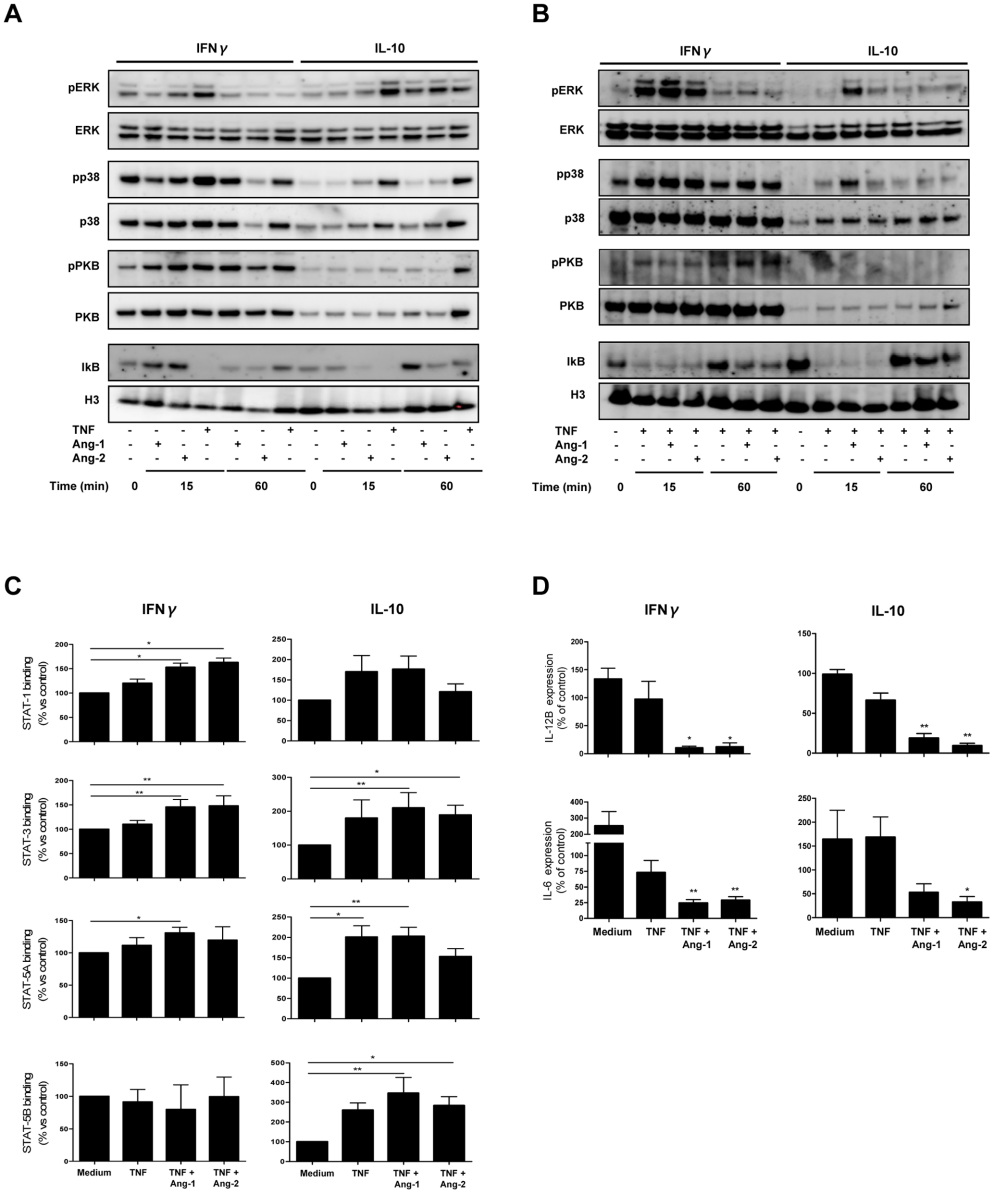
Ang-1 and Ang-2 enhance TNF-induced activation of macrophages via JAK/STAT signaling. (A) Immunoblot analyses of cellular lysates obtained from IFN-γ or IL-10 differentiated macrophages stimulated for the indicated times (minutes) with TNF (10 ng/ml), Ang-1 (200 ng/ml) or Ang-2 (200 ng/ml) for expression and phosphorylation (p) of the indicated signaling proteins. All data shown are from independent immunoblots performed on macrophage lysates obtained from a single donor and are representative of 4 individual experiments. (B) Immunoblot analyses of cellular lysates obtained from IFN-γ or IL-10 differentiated macrophages stimulated for the indicated times (minutes) with TNF (10 ng/ml) in the presence or absence of Ang-1 (200 ng/ml) or Ang-2 (200 ng/ml) for expression and phosphorylation (p) of the indicated signaling proteins. All data shown are from independent immunoblots performed on macrophage lysates obtained from a single donor and are representative of 4 individual experiments. (C) STAT family DNA-binding activity in nuclear lysates obtained from macrophages stimulated for 2 h with TNF (10 ng/ml) in the absence or presence of Ang-1 (200 ng/ml) or Ang-2 (200 ng/ml). Bars represent the means and SEM of 4 independent experiments. *P<0.05, **P<0.01. Friedman test was used for statistical analyses. (D) *IL12B* (upper panels) and *IL6* mRNA (lower panels) expression levels in IFN-γ (left panels) and IL-10 –differentiated macrophages (right panels) after stimulation with medium alone or TNF (10 ng/ml) in the absence or presence of Ang-1 (200 ng/ml) or Ang-2 (200 ng/ml), in the presence or absence of AG490 (10 µM). Values in are represented as percentage mRNA expression in the presence of AG490 in the defined conditions relative to expression in the absence of the inhibitor. Bars represent the means and SEM of 5 independent experiments. *P<0.05, **P<0.01. Friedman test was used for statistical analyses.

To better understand the molecular basis by which Ang-1 and Ang-2 cooperate with TNF to drive gene expression in macrophages, we analyzed activation of key TNF-regulated signaling pathways in polarized macrophages. Ang-1 and Ang-2 alone failed to activate ERK, p38 or PKB in MΦ_IFN_ (Figure 7A). However, Ang-1 and Ang-2 did slightly reduce IκBα expression compared to unstimulated macrophages, although difference were only significant in macrophages stimulated with Ang-1 (P<0.05) (Figure 7A and Figure S2). In MΦ_Il-10_, we found that Ang-1 and Ang-2 induced ERK activation at 60 min, but differences were only significant with Ang-1 (P<0.05) (Figure 7A and S2). Both Ang-1 and Ang-2 failed to modulate activation of p38, PKB or the IkBα expression in MΦ_IL10_. TNF- induced activation of ERK was significantly enhanced (P<0.05), while both Ang-1 and Ang-2 cooperatively induced ERK activation in MΦ_IL10_ (Figure 7B and S3). Ang-1 also extended TNF-dependent p38 activation in both MΦ_IFN_ and MΦ_IL10_ (Figure 7B and Figure S3). No significant impact of Ang-1 or Ang-2 on TNF-induced IkBα degradation or PKB activation was observed in either macrophage population.

As the above experiments failed to give a clear indication as to how Ang-1 and Ang-2 might cooperate with TNF to regulate macrophage gene expression, we next examined a potential role for JAK/STAT signaling. TNF stimulation alone failed to influence activation of STAT-1, -3, -5A and 5B transcription factors in MΦ_IFN_, but Ang-1 and Ang-2, in combination with TNF, significantly induced activation of STAT-1 and STAT-3, and enhanced binding activity of STAT-5A was also observered in the presence of Ang-1 (Figure 7C). In MΦ_IL10_, TNF by itself could stimulate STAT-5A, while Ang-1 and Ang-2 both cooperated with TNF to induce activation of STAT-3 and STAT-5B (Figure 7C). Pharmacological inhibition of JAK/STAT signaling selectively and significantly abrogated contributions of Ang-1 and Ang-2 to macrophage IL-12B and IL-6 mRNA expression, while leaving basal and TNF-induced expression largely intact (Figure 7D). Collectively, these experiments provide evidence that while Ang-1 and Ang-2 similarly and slightly enhance many intracellular pathways common to TNF signaling in MΦ_IFN_ and MΦ_IL10_, unique capacities of Tie2 to promote JAK/STAT activation contribute strongly to cooperative activation of macrophages by Ang-1 and Ang-2.

## Discussion

Myeloid Tie2 signaling plays an essential role in integrating angiogenesis and tissue remodeling to permit the growth of solid tumors [13], [14]. Gene expression profiles have indicated that murine TEMs represent a distinct lineage commitment during monocyte differentiation, and restriction of myeloid Tie2 expression to a distinct subset of circulating human monocytes expressing genes associated with alternatively activated, M2-like macrophages has also been reported [15], [16]. Prominent secreted gene products expressed by human TEMs include cathepsin B, MMP-9, and IL-10, and expression of these factors, important for angiogenesis and immune regulation, is reinforced by Ang-2 stimulation [16], [17], [25]. Also, Ang-2 supports TEM activation of immunosuppressive Tregs, potentiating tumor growth *in vivo*, again indicating an anti-inflammatory function for Tie2 signaling in myeloid cells [17]. However, we now provide direct evidence that human macrophage Tie2 expression is not restricted to cells derived from a distinct, preprogrammed circulating monocyte subset, but instead is highly dependent upon the cytokine milieu present during macrophage differentiation. Tie2 expression is readily detected on pro-inflammatory M1 (IFN-γ, GM-CSF), alternatively activated/wound healing M2 (M-CSF) and immunosuppressive M2 (IL-10)-differentiated macrophages, indicating that Tie2 expression is dissociated from macrophage functional classification.

Although Ang-1 and Ang-2 by themselves have weak to negligible effects on macrophage gene expression, these cytokines interact strongly with TNF to promote gene expression in both M1 and M2 macrophages. Thus, as is the case with ECs, myeloid Tie2 signaling appears to play an important role in integrating angiogenesis with local inflammatory cues [12]. One relevant physiological consequence of macrophage Tie2 stimulation that we identify here is the generation of chemokines which attract peripheral blood monocytes. Disparate studies have previously indicated that Ang-1 and Ang-2 can directly promote monocyte and TEM recruitment by acting as intrinsic chemotactic factors [25], [26]. Although we do observe trends towards monocyte recruitment by Ang-1 and Ang-2 alone, cultured medium from either M1 or M2 macrophages stimulated by Ang-1 or Ang-2 in combination with TNF reproducibly induced monocyte chemotactic responses. Thus *in vivo*, Ang-1 and Ang-2 might perpetuate inflammation both through direct effects on ECs, increasing vascular permeability, as well as stimulating chemokine production by macrophages in the tissue [11].

TEMs, especially within the context of Ang-2 stimulation, display an immunoregulatory phenotype, characterized by low expression of IL-12 and high expression of IL-10 [15]–[17]. In contrast, we find that in both M1 and M2 macrophages, Ang-2 synergizes with TNF to induce IL-12 expression, and by itself can suppress IL-10 production in M2 macrophages. The differences we observe between polarized macrophages and reported data on TEMs may simply reflect differences in gene regulation between monocytes and differentiated macrophages, or intrinsic properties of TEMs as a committed myeloid subpopulation. Remarkably, we find that for most genes studied, Ang-1 synergizes more potently with TNF than Ang-2 to drive macrophage gene expression. In contrast, TEMs are generally unresponsive to Ang-1, although limited effects of Ang-1 on CCL17 suppression and EGF induction have been reported [16]. We find that Ang-1 and Ang-2 cooperate with TNF in a similar fashion to regulate gene expression in both M1 and M2 macrophages. However, we do note that Ang-2 can selectively regulate expression of some genes, such as *CHGA*, *COL4A3*, *IL17F*, *TIE1*, *NPPB*, and *PROK1*, indicating that as yet unidentified signaling pathways may be differentially regulated by Ang-1 and Ang-2.

Importantly, we show that the influence of Ang-1 and Ang-2 on TNF-induced gene expression in macrophages is largely dependent upon activation of JAK/STAT signaling pathways. It has previously been shown that Ang-1 can stimulate phosphorylation of STAT-1, STAT-3, STAT-5 and STAT-6 in human acute myeloid leukemia cells, although the physiological consequences of this were not identified [27]. We observe that Ang-1 and Ang-2 activate STAT-1 and STAT-3 in MΦ_IFN_, and STAT-3 and STAT-5 in MΦ_IL10_. Although we have not assessed the specific contributions of each STAT member to IL-6 and IL-12 production in polarized macrophages, pharmacological inhibition of JAK proteins selectively inhibits the ability of Tie2 to cooperate with TNFR stimulation to regulate these gene products. We also note that both Ang-1 and Ang-2 slightly enhance TNF-dependent ERK and p38 activation. Activation of these kinases might also contribute to the effects of Ang-1 and Ang-2 on macrophage gene expression, either through signaling to additional transcription factors, or more direct interactions with JAK/STAT signaling. In this regard, mitogen-activated protein kinase cascades have been reported to directly phosphorylate STAT1 and STAT3 on serine residues, enhancing their DNA-binding activity [28]–[30]. Alternatively, JAK2 and JAK3 can stimulate ERK activation, providing another possible mechanism of interaction between TNF and Ang-1/Ang-2 signaling [31], [32].

We find that functional expression of Tie2 is a general feature of differentiated macrophages that, although regulated by polarization conditions, is not related to the functional phenotype of the macrophage. The data presented here offer not only an explanation for the dichotomous role of myeloid Tie2 in supporting both immunosuppression in tumor biology and immune activation in RA, but also highlights how specific pathological conditions might contribute to macrophage plasticity [33], [34]. TAM and TEM M2-like functional characteristics are reinforced by tumor-derived cytokines [2], [15]–[17], [35]. Tumor-derived Ang-2, for example, increases TEM IL-10 production, while suppressing IL-12, together directly and indirectly hindering tumor rejection by effector T cells [16], [17]. However, IFN-γ can convert TAMs into M1-like macrophages which promote tumor regression, and it would be of interest to determine if myeloid Tie2 signaling can modulate this effect [36]. We have previously observed Tie2 activation in synovial macrophages of patients with RA and psoriatic arthritis, a form of spondyloarthritis (SpA) [19], and in RA and other forms of arthritis, distinct macrophages with M2-like and mixed M1/M2 phenotypes are observed [20], [22], [37]. In SpA, expression of the M2 marker CD163 is elevated in intimal lining layer macrophages compared to RA, consistent with a relative lack of M1-polarizing cytokines in SpA synovial fluid [37], [38]. Despite this, in both RA and SpA TNF plays a prominent role in driving disease, and synovial macrophage numbers and cytokine products are closely associated with disease activity and patient responses to treatment, indicating contributions to inflammation and joint destruction independent of M1/M2 phenotype [20], [39]–[41]. Our observation that Ang-1 and Ang-2 alone have negligible effects on macrophage polarization and gene expression is reminiscent of studies showing that immune complexes, generally associated with M2 polarization in murine monocytes, also fail to drive human macrophage polarization, while enhancing TLR-induced IL-10 production in both M1 and M2 macrophages [42]. Attempts to understand macrophage plasticity *in vivo* have provided evidence that initiation and resolution of inflammation, and tissue damage and repair, are accompanied by sequential replacement of tissue macrophages with M1 and M2 macrophages [43], [44]. Our data suggest that otherwise neutral “costimulatory” macrophage agonists, such as Ang-1/Ang-2 and immune complexes, accompanied by changing expression of prominent macrophage agonists, such as TNF, IFNγ, TLR ligands, IL-10 and IL-4, may override macrophage phenotypic and functional characteristics to regulate tissue damage and repair.

## Supporting Information

Figure S1 **Ang-1 and Ang-2 enhance the TNF-induced expression of angiogenesis related genes.** (A–D) mRNA expression of angiogenesis related genes in macrophages differentiated with GM-CSF, IFN-γ or IL-10 after 4 h incubation in medium alone or TNF-α (10 ng/ml) in the absence or presence of Ang-1 (200 ng/ml) or Ang-2 (200 ng/ml) (n = 3). Data is shown as relative quantity respect to unstimulated cells, as described in material and methods. Bars represent the means and SEM of 3 independent experiments. *P<0.05, between stimulatory conditions. Friedman test was used for statistical analyses.
(TIF)

Figure S2 **Effects of Ang-1 and Ang-2 on signaling pathways in polarized macrophages.** Data represents densitometric analysis of immunoblots shown in Figure 7A and are shown as relative expression (a.u.) with respect to unstimulated cells, as described in material and methods. Bars represent the means and SEM of 4 independent experiments. #P<0.05, ##P<0.01, compared to medium. Friedman test was used for statistical analyses.
(TIF)

Figure S3 **Effects of Ang-1 and Ang-2 on TNF-dependent signaling pathways in polarized macrophages.** Data represents densitometric analysis of immunoblots shown in Figure 7B and are shown as relative expression (a.u.) with respect to unstimulated cells, as described in material and methods. Bars represent the means and SEM of 4 independent experiments. *P<0.05, between stimulatory conditions, #P<0.05, ##P<0.01, ### P<0.001, compared to unstimulated conditions. Friedman test was used for statistical analyses.
(TIF)

Table S1 Effects of Ang-1 and Ang-2 on macrophage gene expression.
(DOC)
