# Supplementary figures and images for: Tie2 Signaling Cooperates with TNF to Promote the Pro-Inflammatory Activation of Human Macrophages Independently of Macrophage Functional Phenotype
Source: PLoS One. 2014 Jan 3;9(1):e82088. doi: 10.1371/journal.pone.0082088 (PMC3880273; doi:10.1371/journal.pone.0082088)

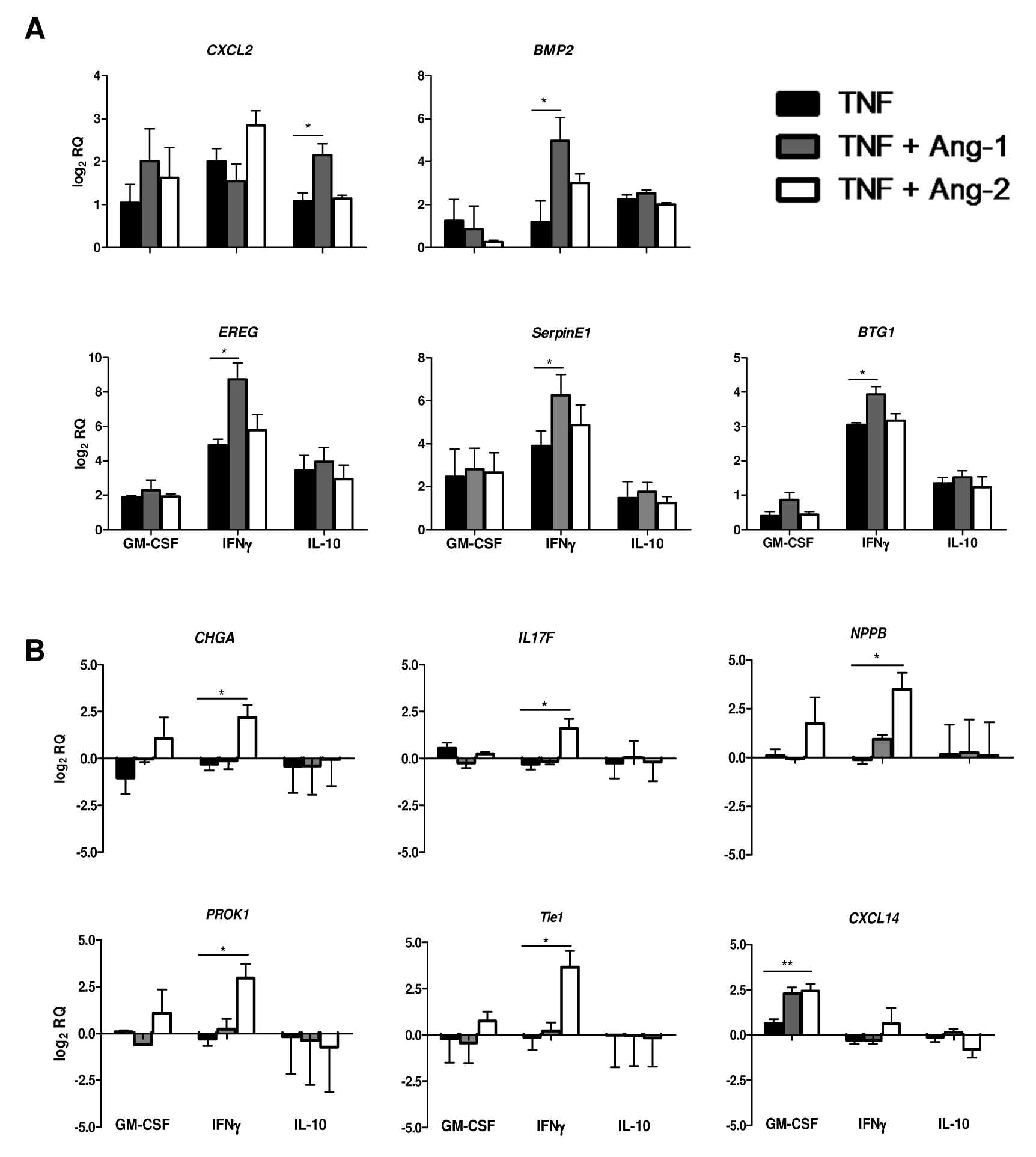

Supplement: Figure S1 — Ang-1 and Ang-2 enhance the TNF-induced expression of angiogenesis related genes. (A–D) mRNA expression of angiogenesis related genes in macrophages differentiated with GM-CSF, IFN-γ or IL-10 after 4 h incubation in medium alone or TNF-α (10 ng/ml) in the absence or presence of Ang-1 (200 ng/ml) or Ang-2 (200 ng/ml) (n = 3). Data is shown as relative quantity respect to unstimulated cells, as described in material and methods. Bars represent the means and SEM of 3 independent experiments. *P<0.05, between stimulatory conditions. Friedman test was used for statistical analyses. (TIF) [file pone.0082088.s001.tif]

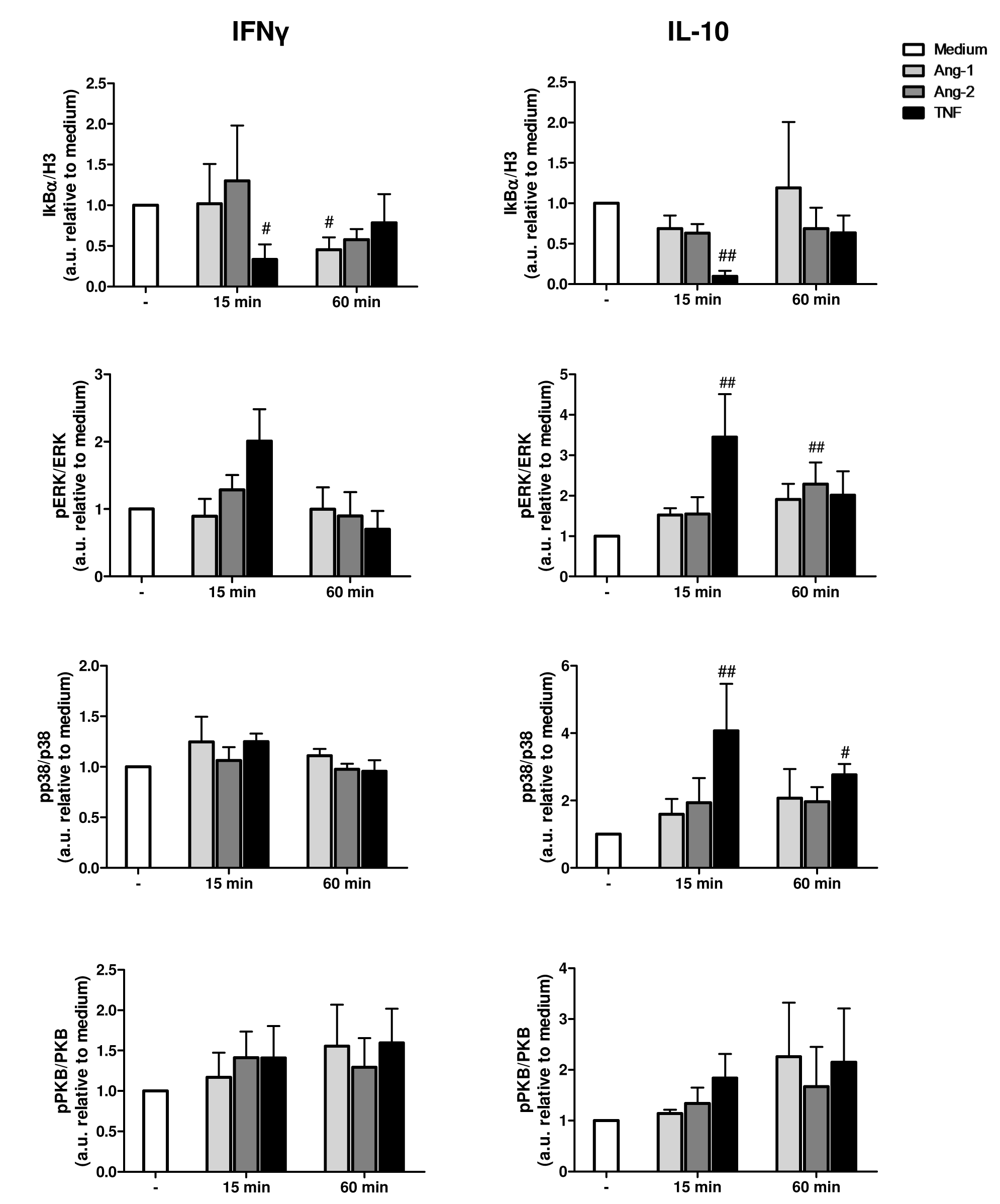

Supplement: Figure S2 — Effects of Ang-1 and Ang-2 on signaling pathways in polarized macrophages. Data represents densitometric analysis of immunoblots shown in Figure 7A and are shown as relative expression (a.u.) with respect to unstimulated cells, as described in material and methods. Bars represent the means and SEM of 4 independent experiments. #P<0.05, ##P<0.01, compared to medium. Friedman test was used for statistical analyses. (TIF) [file pone.0082088.s002.tif]

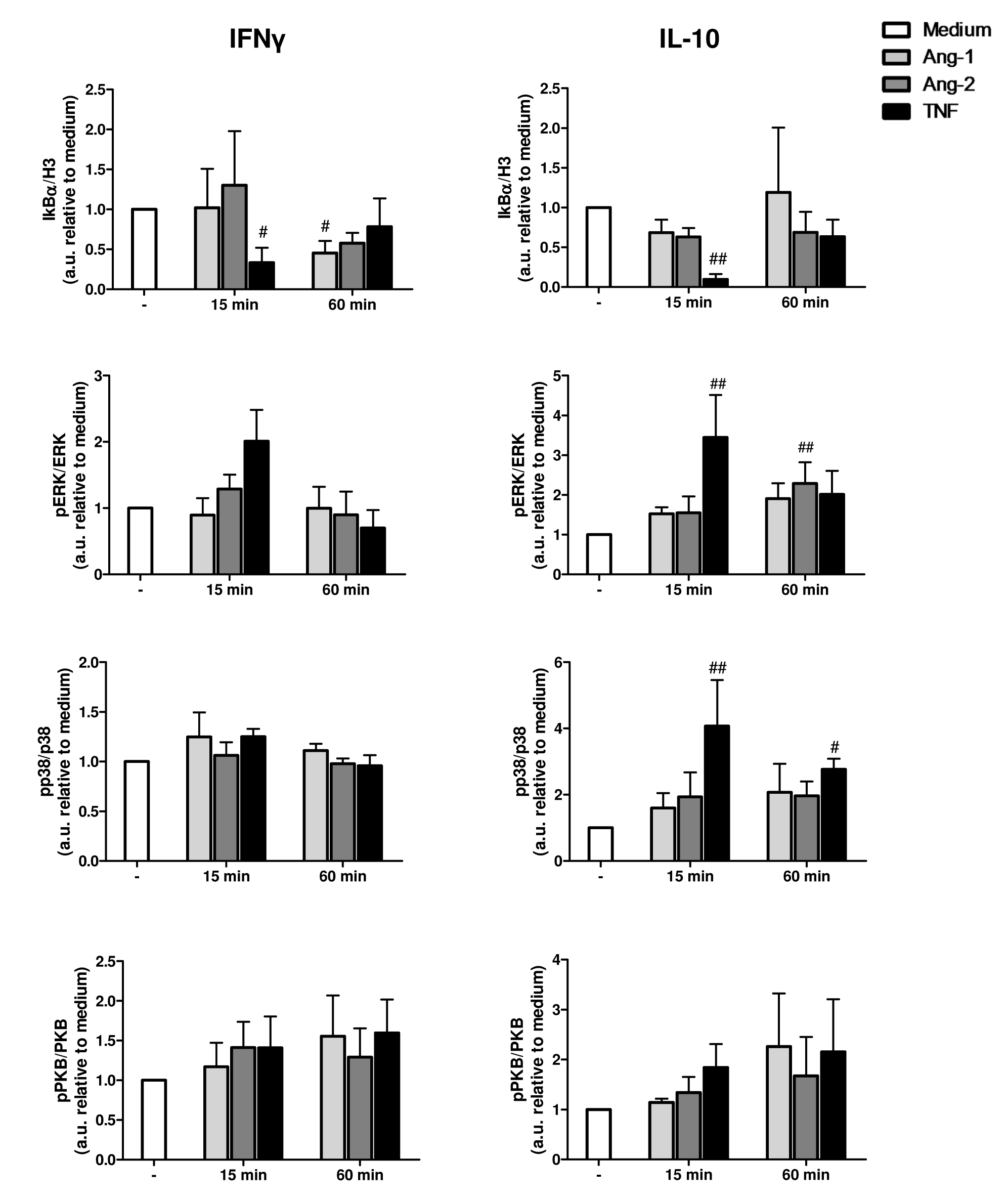

Supplement: Figure S3 — Effects of Ang-1 and Ang-2 on TNF-dependent signaling pathways in polarized macrophages. Data represents densitometric analysis of immunoblots shown in Figure 7B and are shown as relative expression (a.u.) with respect to unstimulated cells, as described in material and methods. Bars represent the means and SEM of 4 independent experiments. *P<0.05, between stimulatory conditions, #P<0.05, ##P<0.01, ### P<0.001, compared to unstimulated conditions. Friedman test was used for statistical analyses. (TIF) [file pone.0082088.s003.tif]
