# Supplementary material for: Tie2 Signaling Cooperates with TNF to Promote the Pro-Inflammatory Activation of Human Macrophages Independently of Macrophage Functional Phenotype
Source: PLoS One. 2014 Jan 3;9(1):e82088. doi: 10.1371/journal.pone.0082088 (PMC3880273; doi:10.1371/journal.pone.0082088)
Supplement: Table S1 — Effects of Ang-1 and Ang-2 on macrophage gene expression. (DOC) [file pone.0082088.s004.doc]

**Table S1. Effects of Ang-1 and Ang-2 on macrophage gene expression**

**Table S1.** Comparison of expression of 84 genes involved in the regulation of angiogenic processes in macrophages differentiated in GM-CSF, IFN-γ, or IL-10 following 4 h incubation in medium alone or TNF (10 ng/ml) in the absence or presence of Ang-1 (200 ng/ml) or Ang-2 (200 ng/ml). Results indicate the RQ in relation to unstimulated cells, as described in materials and methods, and are presented as the mean of 3 independent experiments.
